# The Epidemiology of Syphilis Worldwide in the Last Decade

**DOI:** 10.3390/jcm14155308

**Published:** 2025-07-28

**Authors:** Francois Rosset, Valentina Celoria, Sergio Delmonte, Luca Mastorino, Nadia Sciamarrelli, Sara Boskovic, Simone Ribero, Pietro Quaglino

**Affiliations:** 1 School of Dermatology and Venereology, Department of Medical Sciences, University of Turin, 10123 Turin, Italy; valentinaceloria93@gmail.com (V.C.); nadia.sciamarrelli@gmail.com (N.S.);; 2 Multidisciplinary Centre for Sexual Health (Ce.Mu.S.S.), Department of Prevention, ASL Città di Torino, 10149 Turin, Italy

**Keywords:** syphilis, global health, epidemiology, surveillance, MSM, congenital syphilis, narrative review

## Abstract

**Background/Objectives:** Syphilis, a re-emerging global public health issue, has shown increasing incidence over the past decade, particularly among key populations such as men who have sex with men (MSM), people living with HIV, and pregnant women. This narrative review aimed to synthesize global epidemiological trends of syphilis from 2015 to 2025, with a focus on surveillance gaps, regional disparities, and structural determinants. **Methods:** A broad narrative approach was used to collect and analyze epidemiological data from 2015 to 2025. The literature was retrieved from databases (PubMed, Scopus) and official reports from the WHO, CDC, and ECDC. Included materials span observational studies, surveillance reports, and modeling data relevant to global trends and public health responses. **Results:** Globally, syphilis incidence has increased, with notable surges in North America, Europe, and Asia. MSM remain disproportionately affected, while congenital syphilis is resurging even in high-income countries. Low- and middle-income countries report persistent burdens, especially among women of reproductive age, often exacerbated by limited screening and surveillance infrastructure. The COVID-19 pandemic disrupted syphilis-related services and further exacerbated underreporting, hindering timely detection and response efforts. Surveillance systems vary widely in their completeness and quality, which significantly hinders global data comparability and coordinated public health responses. **Conclusions:** Despite its curability, syphilis continues to spread due to fragmented prevention strategies, inequities in access to care, and insufficient surveillance. Strengthening diagnostic access, integrating prevention efforts into broader health systems, and addressing social determinants are essential. Improved surveillance, equitable access, and innovation—including diagnostics and potential vaccine research—are critical to controlling the global syphilis epidemic.

## 1. Introduction

Syphilis is a sexually transmitted infection (STI) caused by the bacterium *Treponema pallidum* subspecies *pallidum*. First described in detail during the Renaissance period, the disease has long occupied a central role in the history of infectious diseases due to its diverse clinical presentations, its chronic course in untreated individuals, and its intersection with social and sexual behaviors. Historically known as “the great imitator” because of its ability to mimic a wide array of clinical conditions, syphilis was a major public health burden in the early 20th century until the introduction of penicillin, which led to a sharp decline in incidence in most industrialized countries [1,2].

Despite these advances, syphilis has re-emerged globally as a significant public health challenge over the past two decades. Since the early 2000s, several high-income countries have reported a consistent rise in the number of syphilis cases, particularly among MSM, a population disproportionately affected by this infection [3,4]. At the same time, low- and middle-income countries continue to struggle with high rates of congenital syphilis, limited diagnostic access, and incomplete surveillance systems [5]. According to the World Health Organization (WHO), in 2022 alone, an estimated 7.1 million new syphilis infections occurred globally, and congenital syphilis remains a leading cause of stillbirth in many resource-constrained settings [6].

The resurgence of syphilis is influenced by multiple and intersecting factors, including changing sexual networks, reduced condom use, syndemic interactions with HIV, and social determinants such as migration, stigma, and poverty [7]. Furthermore, the COVID-19 pandemic has disrupted many STI services, resulting in reduced screening, treatment delays, and likely underreporting, all of which may have masked or exacerbated transmission trends during this period [8,9]. Although not the primary focus of this review, it is important to acknowledge that the increasing availability of pre-exposure prophylaxis (PrEP) for HIV may influence sexual risk behaviors and indirectly affect syphilis transmission dynamics in some populations [10].

Although syphilis is both curable with benzathine penicillin and preventable through routine screening and behavioral interventions, current public health strategies have not been fully successful in reversing the rising trends observed across many regions. Additionally, persistent disparities in access to diagnostic testing, partner notification, and antenatal screening continue to hinder effective disease control [11,12].

This narrative review provides an updated synthesis of the global epidemiology of syphilis from 2015 to 2025. By examining temporal trends, geographical differences, and the burden among key populations, the review has sought to highlight the most critical challenges in surveillance, prevention, and control. Unlike a systematic review, this work adopted a narrative format to integrate data from a wide range of sources, including epidemiological studies, official health reports, and global surveillance databases. Particular attention has been given to the structural and contextual factors that shape the epidemiology of syphilis, with the goal of informing more equitable and effective responses in the decade ahead. The review has been structured to guide the reader progressively from a global overview of syphilis epidemiology to regional disparities, then to specific populations at higher risk, and finally to surveillance gaps and public health responses. This approach was designed to provide a clear and logical progression from broad trends to targeted insights.

## 2. Materials and Methods

This review adopted a narrative approach to synthesize and interpret epidemiological evidence on the global trends and determinants of syphilis from 2015 to 2025. The scope has been intentionally broad to allow the inclusion of diverse data sources, reflecting the complexity of syphilis epidemiology and the multidimensional nature of its determinants. The main objective was to provide an integrated and critical overview of global patterns, high-risk populations, surveillance systems, and public health responses, with particular attention to regional disparities and recent challenges, including the COVID-19 pandemic.

To gather the relevant literature and surveillance data, a structured yet flexible search strategy was employed. Scientific articles were identified through PubMed and Scopus using combinations of search terms such as “syphilis,” “epidemiology,” “incidence,” “prevalence,” “global,” “congenital syphilis,” “MSM,” and “surveillance.” In addition to standard epidemiological keywords, complementary MeSH terms such as ‘Public Health Surveillance,’ ‘Global Health,’ and ‘Sexual Behavior’ were considered to ensure a broader and more inclusive coverage of the literature. Grey literature and official data were retrieved from the websites of international agencies, including the World Health Organization (WHO), the Centers for Disease Control and Prevention (CDC), and the European Centre for Disease Prevention and Control (ECDC). The time frame considered for inclusion was 2015–2025, with searches performed up to May 2025. Only sources in English were included to ensure consistency and comparability. Although narrative reviews rely on the authors’ interpretation of available evidence, predefined inclusion criteria were applied to enhance the rigor of this synthesis. Priority was given to official surveillance reports from the World Health Organization (WHO), the Centers for Disease Control and Prevention (CDC), and the European Centre for Disease Prevention and Control (ECDC), alongside peer-reviewed epidemiological studies with robust methodology and high regional representativeness. Grey literature and modeling studies were considered only when they offered essential contextual information unavailable in peer-reviewed sources.

Inclusion criteria comprised epidemiological studies (both observational and modeling-based), official surveillance reports, and relevant institutional documents providing quantitative or qualitative insight into syphilis trends or public health strategies. Studies focused solely on clinical or microbiological aspects without population-level data were excluded. Systematic reviews and meta-analyses were also considered when they contributed contextual value to the interpretation of global patterns.

Articles and documents were reviewed and synthesized thematically. This included mapping major trends in incidence and prevalence, identifying geographic and demographic disparities, and highlighting challenges in prevention and control. The synthesis was guided by an iterative reading and comparison of sources, allowing the identification of recurring themes, contradictions, and knowledge gaps. A reflexive approach was maintained throughout to ensure a balanced interpretation of evidence across different settings and population groups.

While this is not a systematic review, efforts were made to ensure methodological transparency and analytical rigor. The review follows general principles outlined in the SANRA (Scale for the Assessment of Narrative Review Articles) checklist, including clarity of aims, relevance of included studies, and critical discussion of findings [13,14,15]. Recognizing the inherent limitations of narrative reviews—such as potential selection bias and lack of reproducibility—this work emphasizes comprehensiveness and coherence in presenting the state of global syphilis epidemiology. Generative Artificial Intelligence (GenAI) tools were employed to assist in structuring the review and in conducting spelling and grammatical revisions.

## 3. Results

### 3.1. Global Epidemiological Trends

**Figure 1:**
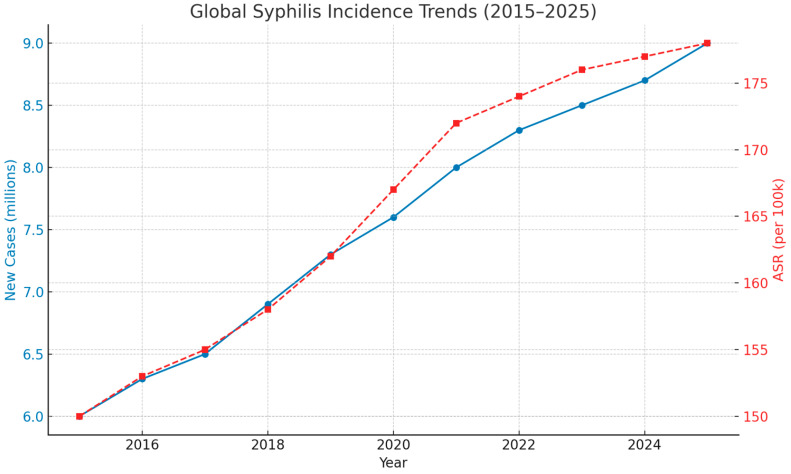
Global Syphilis Incidence Trends, 2015–2025. This dual-axis line chart illustrates estimated global trends in syphilis between 2015 and 2025. The blue line shows the annual number of new syphilis cases (in millions), rising from approximately 6.0 million in 2015 to a projected 9.0 million in 2025. The red dashed line depicts the age-standardized incidence rate (ASR) per 100,000 population, increasing from 150 to 178 during the same period. The age-standardized incidence rate (ASR) was derived from the WHO Global Health Observatory and Global Burden of Disease (GBD) datasets. Rates were standardized using the WHO world standard population to ensure comparability across regions and time periods. These estimates are based on synthesized data from the Global Burden of Disease (GBD) study and the World Health Organization (WHO) Global Health Observatory. The observed trends underscore the persistent and growing global burden of syphilis and the need for reinforced prevention, screening, and treatment strategies.

Over the past decade (2015–2025), syphilis has re-emerged as a global health concern, characterized by complex and divergent epidemiological patterns across continents and population groups [16,17]. According to recent WHO estimates, approximately 8 million new syphilis infections occurred among adults aged 15–49 years in 2022 alone, with an additional 700,000 cases of congenital syphilis globally (Figure 1) [6]. These figures represent a considerable increase compared to the early 2000s, reversing decades of decline and indicating ongoing transmission in both high- and low-resource settings (Table 1).

### 3.2. Global Overview (2015–2025)

Globally, both incidence and prevalence rates of syphilis have been rising. The Global Burden of Disease (GBD) study estimated that in 2015, over 45 million individuals were living with syphilis, with nearly 6 million new infections annually and more than 107,000 attributable deaths [18]. Age-standardized incidence rates have increased since 2010 in numerous countries, with projections suggesting continued elevation or plateauing through 2035, particularly among younger adults [19]. This resurgence has been driven by a combination of behavioral, social, and systemic factors, including changes in sexual networks, reduced condom use, and limited access to sexual health services in many regions.

### 3.3. Continental Distribution and Regional Trends

#### 3.3.1. Europe

In the European Union/European Economic Area (EU/EEA), syphilis notification rates have risen steadily. In 2022, a total of 35,391 confirmed cases were reported, corresponding to a rate of 8.5 per 100,000 population—an increase of 34% from 2021 and 41% from 2018 [4]. The majority of cases were concentrated in Germany, France, the United Kingdom, and Spain. Men accounted for over 85% of cases, and among those with known transmission category, 74% were among MSM [20].

#### 3.3.2. Americas

In North America, particularly the United States, syphilis has reached levels not observed since the 1990s. Primary and secondary syphilis rates increased from 2.1 per 100,000 in 2001 to 17.6 per 100,000 in 2021, with a sharper acceleration observed between 2016 and 2022 [3]. In 2022 alone, the U.S. reported 207,255 total syphilis cases, including 3755 cases of congenital syphilis—the highest number recorded since 1991 [21]. Similarly, in Latin America and the Caribbean, prevalence remains high, with antenatal syphilis affecting up to 2.6% of pregnant women and significantly higher rates observed in marginalized groups such as MSM and sex workers [22].

#### 3.3.3. Africa

Sub-Saharan Africa continues to bear a disproportionate burden of syphilis. WHO data suggest that an estimated 3.5 million women of reproductive age are infected annually in the region [23]. Prevalence among pregnant women ranges from 3% to over 9%, depending on the country, and congenital syphilis remains a major cause of adverse birth outcomes, including stillbirth and neonatal death [24].

#### 3.3.4. Asia and the Pacific

In East and Southeast Asia, syphilis has also resurged in recent years. China, for instance, has experienced a substantial increase in cases among both MSM and heterosexual populations. National surveillance data show that the incidence rate rose from 20.1 per 100,000 in 2010 to 34.5 per 100,000 in 2020, with particular concern regarding increases in urban centers and among young adults [25]. Similar trends have been observed in Russia and parts of Central Asia, where economic instability, migration, and under-resourced health systems contribute to ongoing transmission [26].

## 4. Demographic Trends (Age and Sex)

Globally, syphilis disproportionately affects men. In the United States, in 2022, 83% of primary and secondary syphilis cases were among men, with MSM accounting for the majority of new diagnoses [20]. Among MSM, coinfection with HIV is common; in the U.S., approximately 47% of MSM with syphilis were also living with HIV [26]. Age-specific patterns reveal that the highest incidence rates are typically found in individuals aged 25–34 years, followed by the 20–24 and 35–44-year groups [3,4].

In Europe, the male-to-female ratio among reported syphilis cases was approximately 8:1 in 2022 [4]. In contrast, in sub-Saharan Africa and parts of Latin America, the disease burden among women remains significant, particularly in the context of maternal and congenital syphilis. Pregnant women aged 15–29 years represent a key demographic in many African countries, where routine antenatal screening is often inconsistently implemented or unavailable [23]. Older adults have become an increasingly relevant population in the dynamics of syphilis transmission, warranting greater attention in prevention and screening strategies. Rising life expectancy, access to sexual enhancement therapies, and improved overall health contribute to prolonged sexual activity in later life. However, reduced condom use after menopause or vasectomy, immunosenescence leading to diminished mucosal immunity, and atypical or mild symptoms often misattributed to age-related conditions can delay diagnosis and increase the risk of prolonged infectiousness [24].

## 5. Notable Regional Variations and Emerging Patterns

Several important regional and subpopulation trends have emerged:

### 5.1. MSM:

This group continues to be the most affected population globally. In both Europe and North America, the majority of syphilis cases are among MSM, with ongoing transmission linked to high rates of partner change, substance use during sex (chemsex), and inconsistent condom use [3,20].

### 5.2. Congenital Syphilis:

A resurgence of congenital syphilis has occurred even in high-income countries. In the U.S., the congenital syphilis rate increased by more than 300% between 2015 and 2022 [21]. Contributing factors include missed opportunities for screening during pregnancy, inadequate prenatal care, and challenges in partner notification.

### 5.3. Heterosexual Transmission:

Although historically associated primarily with MSM in recent decades, heterosexual transmission is rising in several regions, including China, Brazil, and Russia [21,25,26,27,28]. This reflects a broadening of the epidemic and suggests the need for expanded prevention strategies targeting diverse sexual networks.

### 5.4. Data Gaps and Underreporting:

In many low- and middle-income countries, especially in Africa and parts of Asia, syphilis is underdiagnosed and underreported due to limited laboratory capacity, stigma, and weak surveillance infrastructure [11,25]. These limitations hinder accurate burden estimation and the design of responsive public health interventions.

## 6. Populations at Higher Risk

The global epidemiology of syphilis is shaped not only by geographic and regional trends but also by disproportionate impact on specific population groups. Social, behavioral, and structural determinants increase vulnerability to syphilis transmission in certain communities, with clear concentration among MSM, sex workers, people living with HIV, migrants, ethnic minorities, and young adults. Understanding the burden in these groups is essential for targeted intervention and effective public health policy. Beyond MSM, sex workers, and individuals living with HIV, several other vulnerable populations require attention. Indigenous communities often experience limited healthcare access and cultural stigma that hinder timely diagnosis and treatment. Incarcerated individuals face elevated risk due to overcrowded conditions, restricted access to preventive services, and limited routine screening within correctional facilities. Adolescents in resource-constrained settings are further disadvantaged by poor sexual health education and a lack of confidential sexual health services. Targeted, context-specific interventions are needed to address the unique challenges faced by these underserved groups [29].

### 6.1. MSM

MSM represent the population most heavily affected by syphilis in many high-income countries. In the United States, more than 55% of all primary and secondary syphilis cases in 2022 were among MSM [3]. Similarly, in the European Union/European Economic Area (EU/EEA), MSM accounted for 74% of syphilis cases with known transmission category, with peaks in urban centers and among individuals aged 25–39 years [4].

Multiple factors contribute to elevated risk among MSM. High partner turnover, engagement in anonymous sex facilitated by dating apps, participation in group sex, and the use of recreational drugs during sex (chemsex) have been associated with increased incidence [30]. Additionally, despite the effectiveness of condom use, there has been a reported decline in condom use among MSM in the context of HIV pre-exposure prophylaxis (PrEP), which—while reducing HIV transmission—does not protect against syphilis [31]. Although less commonly reported, syphilis transmission between women who have sex with women (WSW) is possible through oral-genital contact and the shared use of sexual devices. This highlights the importance of inclusive prevention strategies that address diverse sexual networks.

### 6.2. Sex Workers

Female, male, and transgender sex workers face a high risk of syphilis infection due to frequent partner change, inconsistent condom use (often related to client pressure or economic vulnerability), and structural barriers to healthcare access. In low- and middle-income countries, sex workers often operate in informal or criminalized contexts, which limits their ability to access STI screening, treatment, and prevention services [32].

Surveillance data from sub-Saharan Africa and Southeast Asia indicate syphilis prevalence among sex workers ranging from 2% to over 10%, depending on the setting and access to health services [33]. Even in high-income countries, sex workers face stigma and legal challenges that hinder routine testing and contact tracing [34].

### 6.3. People Living with HIV

There is a well-established bidirectional interaction between syphilis and HIV infection. Syphilitic ulcers facilitate HIV transmission and acquisition, while HIV infection may alter the clinical course of syphilis and complicate its diagnosis [34]. Among MSM with syphilis in the United States, nearly half are co-infected with HIV [35].

Studies show that HIV-positive individuals may be less likely to present with classic symptoms of syphilis and more likely to experience atypical or more severe manifestations, including neurosyphilis [36]. Despite regular follow-up in HIV care, syphilis reinfection rates among people living with HIV remain high, often associated with unprotected sex and limited changes in sexual behavior following initial treatment [37].

### 6.4. Migrants and Ethnic Minorities

Migrants and ethnic minorities face elevated syphilis risk due to a combination of socioeconomic, legal, and cultural barriers. These may include limited access to healthcare, language difficulties, fear of deportation or discrimination, and lower awareness of STI symptoms and services [38].

In Europe, for instance, increased syphilis incidence has been observed in migrant populations, especially from Eastern Europe, North Africa, and sub-Saharan Africa [29]. In North America, African American and Hispanic communities experience higher syphilis rates than non-Hispanic whites, reflecting underlying health inequities and structural racism [39].

In contexts of forced migration and humanitarian crises, conditions such as overcrowded shelters, disrupted health systems, and sexual violence contribute further to vulnerability. However, syphilis surveillance among migrant populations remains inconsistent, with many countries lacking disaggregated data [40].

### 6.5. Young Adults

Young adults (typically defined as individuals aged 15–29) represent another group at high risk of syphilis infection, particularly in regions with emerging epidemics or weak STI education and prevention infrastructure. This group is often characterized by high rates of partner change, inconsistent condom use, and limited STI awareness [41].

In the United States, people aged 20–29 accounted for over 40% of all reported syphilis cases in 2022 [3]. In sub-Saharan Africa, syphilis prevalence among women aged 15–24 remains high, particularly in antenatal care settings [23]. Adolescents and young adults may also face particular stigma and confidentiality concerns that deter them from accessing sexual health services [42].

In some regions, digital platforms have increased sexual networking among youth, which, although empowering in some respects, may facilitate transmission in the absence of targeted public health messaging. Additionally, health literacy around syphilis symptoms and transmission remains low among adolescents globally [43].

### 6.6. Older Adults

Older adults represent a growing at-risk group for syphilis infection. Key contributing factors include: extended sexual activity into later life facilitated by improved health and access to sexual therapies; reduced condom use due to the absence of pregnancy concerns; immunosenescence, which weakens the body’s defenses against Treponema pallidum; and delayed recognition of symptoms, often mistaken for dermatological or arthritic conditions. These factors collectively increase the risk of transmission and complicate timely treatment in this population [44].

## 7. Surveillance Systems and Data Gaps

Effective surveillance is central to understanding, preventing, and controlling the global spread of syphilis. Despite efforts by international health agencies and national governments, current surveillance systems remain heterogeneous in structure, coverage, and data quality. While countries with established public health infrastructure have made significant progress in case reporting and trend monitoring, many low- and middle-income countries face persistent gaps in data collection, limiting their ability to respond appropriately to the evolving epidemic (Table 2). In addition to sub-Saharan Africa and Southeast Asia, Latin America and the Caribbean remain regions with notable antenatal syphilis prevalence. Brazil, for example, has reported rates exceeding 2.6% among pregnant women, while Haiti continues to experience one of the highest congenital syphilis incidences in the region. These disparities underscore the need for tailored regional strategies.

### 7.1. International Frameworks and Institutional Systems

The World Health Organization (WHO), the Centers for Disease Control and Prevention (CDC), and the European Centre for Disease Prevention and Control (ECDC) each play a central role in global and regional syphilis surveillance. WHO operates through the Global Health Observatory and its Global STI Surveillance program, which compiles data on syphilis incidence, prevalence, and congenital cases, submitted voluntarily by member states [45]. WHO also supports the elimination of mother-to-child transmission (EMTCT) of syphilis through global reporting tools integrated into reproductive and maternal health programs [46].

The CDC in the United States has developed one of the most robust surveillance systems, through the National Notifiable Diseases Surveillance System (NNDSS), which requires mandatory reporting of primary, secondary, early latent, and congenital syphilis cases [16]. The CDC provides real-time epidemiological data disaggregated by age, sex, race/ethnicity, and transmission category, enabling targeted interventions and policy adjustments.

Similarly, ECDC coordinates STI surveillance across EU/EEA countries. National public health institutes report annually to ECDC using standardized case definitions. However, there remain inconsistencies in data completeness and testing strategies between member states, limiting comparability [47]. ECDC reports also reveal that nearly 25% of syphilis cases lack information on transmission mode, and 35% lack data on HIV status, underscoring challenges in surveillance precision [48].

### 7.2. Established vs. Fragmented Systems

Countries with consolidated STI surveillance systems—such as the United States, Canada, Germany, and the United Kingdom—typically benefit from centralized databases, universal healthcare access, and strong reporting mandates. These systems allow for early detection of outbreaks, identification of emerging trends, and integration of testing data from both public and private sectors [49].

In contrast, many low-resource settings—particularly in sub-Saharan Africa, South Asia, and parts of Latin America—rely on sentinel site surveillance, occasional prevalence surveys, or estimates derived from modeling studies due to limited infrastructure [50]. This results in underreporting, incomplete geographical coverage, and delayed data submission. For instance, a 2022 WHO evaluation found that fewer than half of low-income countries regularly submitted syphilis surveillance data, and many lacked disaggregated indicators for sex, age, or pregnancy status [51].

### 7.3. Data Quality, Accessibility, and Interoperability

A major limitation in current global surveillance lies in the quality and interoperability of collected data. Inconsistent case definitions, varying diagnostic standards (e.g., reliance on rapid tests vs. confirmatory serology), and differences in testing frequency hinder international comparability [52]. Moreover, in some regions, data from private healthcare providers or community-based testing programs are not routinely reported to national health systems, resulting in substantial surveillance blind spots [53].

Digitalization has improved surveillance capabilities in some contexts, particularly through the use of electronic health records (EHRs) and centralized STI registries. However, data sharing across jurisdictions remains constrained by privacy laws, technical incompatibilities, and political barriers [54]. Interoperability between STI, HIV, and maternal health databases is also limited, which hampers efforts to monitor syndemic trends and coordinate integrated responses.

Ultimately, strengthening global syphilis surveillance will require investments in laboratory infrastructure, workforce training, and harmonization of indicators. Enhanced coordination between multilateral agencies and national health authorities is also essential to ensure timely data collection, reporting, and use in public health decision-making.

## 8. Impact of COVID-19 on Syphilis Surveillance and Transmission

The COVID-19 pandemic has had a profound and multifaceted impact on health systems worldwide, including the surveillance, diagnosis, and management of sexually transmitted infections (STIs) such as syphilis. Although the direct virological interaction between SARS-CoV-2 and *Treponema pallidum* is negligible, the pandemic has disrupted healthcare services, shifted public health priorities, and altered sexual behavior, contributing both directly and indirectly to changes in syphilis epidemiology.

### 8.1. Disruption of Screening, Diagnosis, and Treatment

During the peak of the pandemic in 2020 and 2021, STI services in many countries were deprioritized or suspended altogether. Routine syphilis screening, particularly in antenatal care and sexual health clinics, was significantly reduced due to clinic closures, staff reassignments, supply chain disruptions, and restrictions on movement [55]. In the United States, the number of syphilis tests performed declined markedly in early 2020, with a concurrent decrease in reported diagnoses—likely reflecting underdetection rather than true declines in incidence [8].

Delays in diagnosis and treatment led to increased risk of onward transmission and more advanced clinical presentations. In the context of congenital syphilis, missed opportunities for early detection in pregnant individuals translated into a rise in adverse outcomes, including stillbirth and neonatal infection [21].

### 8.2. Changes in Sexual Behavior and Risk Dynamics

The pandemic also influenced sexual behavior in complex and context-dependent ways. While early lockdowns and physical distancing measures initially reduced sexual encounters outside of cohabiting relationships, later phases saw a rebound, often accompanied by a relaxation in preventive behaviors such as condom use [56]. Several studies among MSM populations documented reduced access to testing and care, combined with a resurgence in high-risk practices as restrictions eased [57].

The psychological toll of isolation, increased use of dating apps, and shifts toward private or unregulated spaces for sexual encounters (e.g., home settings, group events) may have also contributed to changes in transmission dynamics, especially among younger adults and urban populations [58].

### 8.3. Interruption in Surveillance and Information Flows

Surveillance systems were also affected by the pandemic. Many public health laboratories were repurposed for COVID-19 testing, and routine data reporting on STIs was delayed or interrupted [59]. These gaps compromised trend analyses and hampered timely public health responses. In Europe, ECDC reported that several member states experienced temporary suspensions in STI case reporting in 2020–2021, with substantial delays in data availability for syphilis and other notifiable infections [60].

Additionally, research efforts and funding streams were redirected toward COVID-19, slowing the advancement of syphilis diagnostics, vaccine development, and programmatic innovation. In low- and middle-income countries, the pandemic exacerbated pre-existing weaknesses in STI control programs, further widening global disparities in syphilis detection and care [61].

## 9. Public Health Responses and Challenges

Efforts to control syphilis globally have intensified over the past decade, yet significant public health challenges persist. Although the infection is curable, and diagnostic tools are widely available, gaps in prevention strategies, testing access, and health equity continue to fuel transmission. In parallel, emerging threats such as antibiotic resistance and the absence of a vaccine highlight the need for renewed innovation and investment.

### 9.1. Current Prevention Strategies

Public health strategies to prevent syphilis transmission rely primarily on early detection, timely treatment, partner notification, and behavioral interventions. In many countries, routine screening is recommended for key populations, including MSM, sex workers, people living with HIV, and pregnant women [62]. Integration of syphilis testing into HIV and reproductive health services has proven effective in enhancing coverage and uptake [63].

Educational campaigns aimed at reducing stigma, promoting safer sex practices, and increasing testing awareness remain critical. However, the impact of these campaigns is often limited by structural and cultural barriers, particularly in low- and middle-income countries. Where criminalization or discrimination persists—e.g., against LGBTQ+ populations or sex workers—individuals may avoid testing and care due to fear of social or legal repercussions [64]. Recent reports have also highlighted emerging clinical presentations of syphilis, such as ophthalmological involvement, which add further complexity to its diagnosis and management [62].

### 9.2. Access to Testing and Treatment

Despite the availability of reliable diagnostics, access to syphilis testing is uneven across and within countries. Rapid diagnostic tests (RDTs), particularly those that combine syphilis and HIV detection, offer scalable, point-of-care options that do not require laboratory infrastructure [65]. These tests have been successfully implemented in antenatal settings and mobile outreach programs, but coverage remains far from universal.

The standard treatment for syphilis—intramuscular benzathine penicillin G—remains highly effective for all stages of infection. However, supply chain disruptions and manufacturing shortages have periodically limited global availability, particularly in low-resource settings [66]. Inadequate training of health workers, insufficient awareness among patients, and delays in partner notification further weaken treatment outcomes.

Congenital syphilis prevention requires a system-wide response: universal antenatal screening, immediate treatment of seropositive pregnant women, and follow-up of neonates. Yet, millions of pregnancies occur annually without adequate syphilis screening, leading to preventable perinatal morbidity and mortality [67].

### 9.3. Potential Innovations

Innovation in syphilis prevention and management has been comparatively modest relative to other STIs. Nonetheless, several promising developments have emerged in recent years. Advances in multiplex point-of-care diagnostics allow simultaneous screening for syphilis, HIV, and hepatitis B, improving efficiency and integration of services [68].

Research into a syphilis vaccine is ongoing but remains in preclinical stages. A significant challenge lies in the complex structure of *T. pallidum* and its ability to evade immune responses. However, genomic sequencing of the bacterium and identification of surface-exposed antigens have opened new avenues for immunological targeting [69].

Digital health interventions, including mobile phone reminders, telehealth consultations, and digital partner notification platforms, are also being explored to expand the reach and efficiency of syphilis services [70].

### 9.4. Emerging Risks: Antimicrobial Resistance

Although penicillin remains effective, resistance to alternative treatments—such as macrolides—has been increasingly reported. Strains of *T. pallidum* with mutations conferring resistance to azithromycin have been documented in multiple regions, including the United States, China, and Europe [71]. These findings underscore the importance of continued surveillance and the avoidance of monotherapy with macrolides in settings where resistance is prevalent.

The global reliance on a single antibiotic (penicillin) also poses a strategic vulnerability. If production issues or global supply constraints arise, countries with limited domestic manufacturing capacity may face treatment shortages. Maintaining and diversifying production sources, training providers in injection safety, and ensuring proper dosing are therefore essential components of treatment security [65,72].

## 10. Conclusions

Despite being a preventable and curable infection, syphilis continues to pose a significant threat to global public health. Over the past decade, the epidemiological resurgence of syphilis in both high-income and low-resource settings has underscored systemic failures in prevention, surveillance, and treatment. This narrative review highlights a troubling combination of rising incidence, persistent inequities in health access, and emerging biological and structural threats. Unlike systematic reviews or meta-analyses, this narrative review did not follow a predefined and rigid protocol for literature search, selection, and synthesis. While the narrative approach allows for a broader thematic exploration and contextual discussion, it inevitably introduces a higher degree of subjectivity and potential selection bias. For this reason, the findings presented should be interpreted with caution, recognizing the inherent methodological limitations of narrative reviews.

From 2015 to 2025, the global burden of syphilis has increased across most regions, with particularly sharp rises observed among MSM, people living with HIV, sex workers, and marginalized communities. The re-emergence of congenital syphilis in high-income countries—once thought to be eliminated—alongside persistently high rates in sub-Saharan Africa and Latin America exemplifies the failure of health systems to reach vulnerable populations effectively.

Current public health responses remain fragmented. Although international frameworks (e.g., WHO’s EMTCT initiative) and national STI programs have contributed to progress, implementation varies widely. Structural challenges—such as healthcare access, stigma, criminalization, and insufficient funding—continue to undermine efforts, particularly in resource-limited settings. Inadequate surveillance capacity further limits the ability of governments and agencies to understand the full scope of the epidemic, respond rapidly to outbreaks, or allocate resources efficiently. Comparing syphilis data across countries remains challenging due to substantial differences in surveillance infrastructure, diagnostic standards, and reporting protocols. These inconsistencies lead to data gaps and limit the ability to draw definitive conclusions about global syphilis trends. A move toward more standardized global reporting frameworks would greatly improve the reliability of cross-country comparisons. The reliability of the synthesized evidence is influenced by the heterogeneity in data quality and reporting practices across different regions. While high-income countries generally benefit from well-established surveillance systems and mandatory reporting, many low- and middle-income countries face substantial structural challenges, including limited laboratory capacity, fragmented health information systems, and underreporting. These disparities affect the comparability and accuracy of the global trends presented in this review. The COVID-19 pandemic disrupted routine surveillance systems worldwide, causing significant gaps in data collection and delays in reporting for sexually transmitted infections, including syphilis. Although this review acknowledges the pandemic’s impact, it is likely that the current estimates do not fully reflect the long-term effects of these disruptions on syphilis epidemiology and control efforts.

While this review provides a broad overview of global epidemiological trends, it does not delve deeply into the complex local determinants of syphilis transmission, such as sociocultural norms, political priorities, or economic barriers. These factors can substantially influence health-seeking behaviors, access to services, and the effectiveness of control measures in specific contexts. Further localized studies are essential to better understand these dynamics and design context-appropriate interventions.

## 11. Future Perspectives

Despite recent efforts, the global resurgence of syphilis highlights persistent gaps in surveillance, prevention, and access to care. To effectively curb transmission and reduce disparities, a comprehensive and forward-looking public health strategy must prioritize the following key areas over the coming decade:

1. **Strengthening Surveillance Systems.** National and global surveillance mechanisms need to be expanded and standardized, with greater investment in digital infrastructure, laboratory capacity, and integration with HIV and reproductive health programs.
2. **Ensuring Equitable Access to Diagnostics and Treatment.** Universal availability of point-of-care testing and uninterrupted supplies of benzathine penicillin are essential to ensure timely diagnosis and cure. Special attention must be given to antenatal care and rural or underserved communities.
3. **Expanding Prevention Strategies.** Targeted interventions for key populations—including MSM, migrants, and youth—should be reinforced through community engagement, digital outreach, and peer-led models. Integration of syphilis prevention into sexual education and broader public health campaigns is also needed.
4. **Fostering Innovation.** Investment in new technologies, including rapid multiplex diagnostics and vaccine development, should be accelerated. Genomic surveillance and resistance monitoring will be critical to guide future therapeutic strategies and address emerging antimicrobial resistance.
5. **Addressing Social Determinants.** Long-term success in syphilis control will depend on confronting the social and political determinants of health, such as poverty, stigma, gender-based violence, and the legal environment. Multisectoral approaches are needed to dismantle barriers to care and promote health equity.
6. **Reducing underreporting.** Persistent underreporting in low- and middle-income countries remains a major barrier to accurately assessing the true burden of syphilis. Addressing this gap will require harmonized case definitions, improved laboratory and diagnostic capacity, and the implementation of standardized digital surveillance platforms. Targeted investments in workforce training, data-sharing mechanisms, and antenatal screening programs are crucial steps toward improving surveillance quality and enabling more effective public health responses.

In conclusion, syphilis remains emblematic of broader challenges in global health governance and health system equity. With adequate political will, scientific innovation, and coordinated international support, the burden of syphilis can be significantly reduced in the coming decade. However, this will require shifting from reactive control to proactive, structural, and inclusive approaches.

## Figures and Tables

**Table 1:** Reported syphilis cases, notification rates, and key notes by continent/region (2022).

Continent/Region	Reported Cases (2022)	Notification Rate (per 100,000)	Notes
EU/EEA	35,391	8.5	+34% vs. 2021 (ECDC)
Americas—USA	207,255 total; 3755 congenital	~62.5 (approx.)	Primary/secondary ≈ 17.6/100,000
Americas—Latin America and Caribbean	Data varies by country	Often > 2% antenatal prevalence	Brazil > 2.6% antenatal prevalence; Haiti high congenital rates
Africa—sub-Saharan	n/a (surveillance gaps)	Often > 3% antenatal prevalence	Significant data gaps in many countries
Asia—China	Wuhan example available	~34.5 per 100,000	National incidence continues to rise

**Table 2:** Antenatal care testing, treatment coverage, and congenital syphilis rates by region.

Region	% ANC (Antenatal Care) Attendees Tested	% Treated (if Positive)	Congenital Cases per 100k Births
Africa (median)	50–80%	65–90%	Up to 2000 (e.g., Nigeria)
Latin America	70–95%	80–95%	~100 per 100k (e.g., 2022 in PAHO)
Europe/USA	>95%	>98%	USA congenital 102.5/100k (2022)
